# Pulmonary embolism following rotator cuff repair

**DOI:** 10.4103/0973-6042.42576

**Published:** 2008

**Authors:** Samuel C. Hoxie, John W. Sperling, Robert H. Cofield

**Affiliations:** Department of Orthopedic Surgery, Mayo Clinic, 200 First Street, S.W., Rochester, MN, USA

**Keywords:** Pulmonary embolism, rotator cuff repair, shoulder surgery

## Abstract

**Background:**

Previous studies have provided data on the incidence of pulmonary embolism following shoulder arthroplasty and repair of fractures of the proximal humerus. However, there is no information on the risk of pulmonary embolism following the surgical management of rotator cuff tears.

**Methods:**

We performed a review of 1176 patients who underwent operative procedures for rotator cuff tears between January 1^st^, 2001 and December 31^st^, 2005 to identify all patients who developed a symptomatic pulmonary embolism postoperatively.

**Results:**

Three patients developed pulmonary embolisms that were diagnosed with computed tomography angiography. The overall incidence was calculated to be 0.26%. None of the patients died as a result of the pulmonary embolism.

**Conclusions:**

The data from this review indicates that the risk of pulmonary embolism following surgery for rotator cuff repair is low, but not nonexistent. The most common presenting symptoms of pulmonary embolism were chest pain, shortness of breath, and hypoxia. This study should raise surgeons' awareness about this possible complication following rotator cuff repair surgery.

## INTRODUCTION

Pulmonary embolism is a complication that can follow orthopedic procedures. Studies have defined the risk factors for the development of pulmonary embolism, provided data on the incidence, and determined the probability of death resulting from a pulmonary embolism following many types of procedures. Previous studies have shown that the rate of pulmonary embolism after shoulder surgery varies from 0.17% for elective shoulder arthroplasty to 5.1% after proximal humerus fracture repair (review series from our institution; publication pending). However, there is no information available on the risk of pulmonary embolism following the surgical management of rotator cuff pathology. The purpose of this study was to determine the incidence of pulmonary embolism after the operative treatment of rotator cuff tears.[1]

## MATERIALS AND METHODS

We obtained the approval of the Institutional Review Board before beginning the review. Patients were identified by the use of a surgical database. Consent for the use of information from the medical records was obtained from all patients in the study group. All patients over the age of 16 who underwent operative treatment for rotator cuff tears within the study period were included in the study. No patient who met the inclusion criteria was excluded.

From January 1^st^, 2001 to December 31^st^, 2005, 1176 patients treated surgically for rotator cuff tears were identified. A search of the institution's medical diagnosis index was performed to identify any patient in the study group who was ever diagnosed with, or suspected of having, a pulmonary embolism. A detailed review of the medical records identified the patients who had had a pulmonary embolism within the first 6 weeks following surgery. The complete medical records of all patients diagnosed with a pulmonary embolism were reviewed.

The diagnosis of pulmonary embolism was confirmed by CT angiography in each case. Previous studies have shown that CT angiography is an acceptable modality for the diagnosis of pulmonary embolism.[2–4]

## RESULTS

Of the 1176 patients who underwent surgery for rotator cuff tears, 462 (39.3%) were female and 714 (60.7%) were male. Open surgical procedures were done in 867 (73.7%) cases, and arthroscopic procedures were performed in 309 (26.3%). The mean age of patients undergoing surgery was 60.3 years (range: 16–94); the mean age for women was 62.7 years and for men it was 59.8 years.

Chemoprophylaxis against deep venous thrombosis (DVT) and pulmonary embolism was not routinely used. However, all patients received standard thromboembolic device (TED) hose. Some patients were given sequential compression devices postoperatively at the discretion of the senior surgeon.

Postoperative physical therapy was initiated on the first day following surgery. Patients were instructed to follow a passive motion program for the first 6 weeks following surgery. For the seventh to twelfth postoperative weeks, patients were involved in a home program of active assisted motion, with no heavy lifting. Return to activities without restriction was recommended at 3 months after surgery.

Following surgery, three of the 1176 patients in the study group were diagnosed with a pulmonary embolism, giving an incidence in this series of 0.26%.

One patient was a 63-year-old retired male with a medical history that included coronary artery disease, rheumatoid arthritis, hypertension, and type 2 diabetes mellitus. He underwent an uncomplicated arthroscopic rotator cuff repair. Approximately 30 days following surgery, the patient developed substernal chest pain and shortness of breath. A thorough evaluation excluded cardiac causes for the chest pain. The pain persisted and on the 35^th^  postoperative day, a chest CT revealed bilateral pulmonary emboli. Venous ultrasound showed occlusive thrombus in the right femoral and popliteal veins., Intravenous heparin was used for anticoagulation initially; later the patient was prescribed oral warfarin. Warfarin therapy was continued for 6 months following the diagnosis of pulmonary embolism. This patient has had no further thromboembolic events to date during his 35 months of follow-up.

The second patient was a 52-year-old male who was in good general health, with mild hypertension being the only medical problem present. He underwent an open rotator cuff repair. On postoperative day number one, he developed hypoxia and shortness of breath. A pulmonary embolism was diagnosed on CT scan. Intravenous heparin was used until the patient was therapeutic with oral warfarin. Following the advice of our vascular medicine colleagues, the patient was prescribed 12 months of warfarin anticoagulation therapy and has had no further complications over 30.8 months of follow-up.

The third patient was a 63-year-old male with a history of heterozygote factor V Leiden gene mutation and irritable bowel syndrome. An arthroscopic rotator cuff repair was performed without incident. On postoperative day number seven, the patient had sudden onset of chest pain. Evaluation demonstrated a pulmonary embolism. He was initially treated with intravenous heparin and then advised 6 months of oral warfarin. No further episode of DVT or pulmonary embolism occurred over the subsequent 6.8 months. During the third month of warfarin treatment, the patient sustained a scalp laceration following a standing-height fall. The laceration was evaluated and repaired in the emergency department. The amount of blood lost was likely significantly increased because of the use of oral anticoagulation.

No patient died of pulmonary embolism. In addition, none of the patients developed any major complication due to anticoagulation therapy. The most common presenting symptoms of pulmonary embolism were chest pain, shortness of breath, and hypoxia.

## DISCUSSION

Pulmonary embolism is a known complication following many types of surgical procedures. Several series have reported on shoulder surgeries that have been complicated by thromboembolic events.[1,5–7] However, there is no information available regarding pulmonary embolism following elective rotator cuff repair procedures. This study demonstrates that pulmonary emboli after rotator cuff repair do occur.

In this series, two of the three patients had medical conditions which may have contributed the development of pulmonary embolism. One patient was being treated for rheumatoid arthritis and the second tested positive for heterozygote factor V Leiden gene mutation, which is a known hypercoagulable condition. Both of these medical diagnoses are known to raise the risk of developing thromboembolic events such as pulmonary embolism.

This study should serve to heighten surgeons' awareness regarding pulmonary embolism as a possible complication of operative treatment of rotator cuff tears. Chest pain, shortness of breath, and hypoxia were the most common symptoms of pulmonary embolism. When these symptoms are identified in patients following rotator cuff repair, surgeons should consider the possibility of pulmonary embolism.

We rigorously question patients to try and identify patients at risk for thromboembolic complications following shoulder surgery. Our current treatment protocol begins with obtaining a detailed medical history before surgery. Patients without history of thromboembolic disease or any other risk factor for pulmonary embolism are considered low risk. Postoperatively, these patients receive standard TED elastic support stockings, early ambulatory mobilization, and no chemoprophylaxis for DVT or pulmonary embolism. Patients with a history of thromboembolic disease or other risk factors are considered high risk. Following surgery, these individuals receive sequential compression devices in addition to the standard TED stockings, early mobilization, and a short course of thromboembolic chemoprophylaxis, such as warfarin or low molecular weight heparin.

This study highlights the issue of the use of routine prophylactic anticoagulation in the perioperative period, particularly for high-risk patients. However, the use of anticoagulant medications is not without risk. In this series, only one minor adverse event and no major complications occurred in the three patients because of the use of intravenous or oral anticoagulation, but serious complications from these therapies can also occur. The possibility of anticoagulation-induced increased bleeding episodes, specifically surgical-site hematoma formation, should be carefully considered when initiating these therapies.
